# Integrated Mixed Potential Gas Sensor with Efficient Structure for Discriminative Volatile Organic Compounds Detection

**DOI:** 10.1002/advs.202405124

**Published:** 2024-07-23

**Authors:** Siyuan Lv, Tianyi Gu, Qi Pu, Bin Wang, Xiaoteng Jia, Peng Sun, Lijun Wang, Fangmeng Liu, Geyu Lu

**Affiliations:** ^1^ State Key Laboratory of Integrated Optoelectronics College of Electronic Science and Engineering Jilin University 2699 Qianjin Street Changchun 130012 P. R. China; ^2^ International Center of Future Science Jilin University 2699 Qianjin Street Changchun 130012 P. R. China

**Keywords:** feature engineering, integrated gas sensor, new device structure, pattern recognition, volatile organic compounds detection

## Abstract

Amid growing interest in the precise detection of volatile organic compounds (VOCs) in industrial field, the demand for highly effective gas sensors is at an all‐time high. However, traditional sensors with their classic single‐output signal, bulky and complex integrated structure when forming array often involve complicated technology and high cost, limiting their widespread adoption. Here, this study introduces a novel approach, employing an integrated YSZ‐based (YSZ: yttria‐stabilized zirconia) mixed potential sensor equipped with a triple‐sensing electrode array, to efficiently detect and differentiate six types of VOCs gases. This innovative sensor integrates NiSb_2_O_6_, CuSb_2_O_6_, and MgSb_2_O_6_ sensing electrodes (SEs), which are sensitive to pentane, isoprene, n‐propanol, acetone, acetic acid, and formaldehyde gases. Through feature engineering based on intuitive spike‐based response values, it accentuates the distinct characteristics of every gas. Eventually, an average classification accuracy of 98.8% and an overall R‐squared error (R^2^) of 99.3% for concentration regression toward six target gases can be achieved, showcasing the potential to quantitatively distinguish between industrial hazardous VOCs gases.

## Graphical abstract

An integrated YSZ‐based (YSZ: yttria‐stabilized zirconia) mixed potential sensor equipped with a triple‐sensing electrode array combining with machine learning of feature engineering is utilized to efficiently realize detection and identification between six types of VOCs gases. Ultimately, the qualitative classification accuracy of 98.8% and the overall R^2^ error for concentration regression of 99.3% can be achieved.

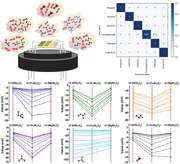

## 1 Introduction

With the advancement of industrial modernization, the variety of volatile organic compounds (VOCs) found in industrial production is on the rise, necessitating enhanced capabilities for VOCs detection. As sensing technology evolves, leading to the development of sensor arrays that mimic human olfaction by employing multiple sensors to differentiate between gases.^[^ ^1^ ^]^ This technological evolution is rooted in the principle that human olfaction discerns various VOCs through numerous receptors. A concept now mirrored in gas detection through sensor arrays combined with pattern recognition algorithms.^[^ ^2^ ^]^

As for gas pattern recognition, current research can be carried out from two aspects: the development of sensor diversity,^[^ ^3^ ^]^ and the enhancement of data processing capability through machine learning (ML).^[^ ^4^ ^]^ Traditionally, the sensor array with large number of high‐performance sensing elements enhances discrimination capabilities. With regard to array consisting of standalone sensors, Sun et al. developed a sensor array to identify various VOCs to detect different bacteria in wound infection, achieving an optimized recognition rate of 95.19% with 10 sensors.^[^ ^5^ ^]^ From the perspective of the multivariable integrated sensor array, Kang et al. designed uniform integrated gas sensor arrays through glancing angle deposition for gas detection between CO, NH_3_, NO_2_, CH_4_, and C_3_H_6_O.^[^ ^3a^ ^]^ Capman et al. utilized graphene‐based gas sensor arrays that each array contains 108 sensors for classification of 5 VOCs species with 4 concentrations each.^[^ ^3b^ ^]^ However, when the number of sensing element increases, it will inevitably face the bottleneck challenge of large size, complex technology or fragile sensing system. Therefore, beyond the development of variety of gas sensors, leveraging varied signal processing for machine learning enhances discrimination efficiency. Ji et al. proposed a multicomponent gas detection method based on the temperature‐response relationship.^[^ ^4c^ ^]^ The multicomponent gas could be recognized with the help of coordinate system transformation and rational Taylor function fitting. Wang et al. applied different bias voltage on the sensor to obtain diverse signal curves.^[^ ^6^ ^]^ Fourier transformation and integral of the signal curves were adopted for discrimination toward 5 different VOCs gases. While in some studies, complex signal conversion or additional time‐consuming method for effective feature extraction is still inescapable. Therefore, to sum up, realizing high sensing and discrimination efficiency with minimum number of sensors and further optimization of data processing is indispensable but challenging.

For the design of highly efficient and compact integrated sensor, among various gas sensors based on different sensing mechanisms,^[^ ^7^ ^]^ the YSZ‐based (YSZ: yttria‐stabilized zirconia) mixed potential type sensors have attracted much attention. As a representative electrochemical sensor, it possesses fast response and recovery speeds, simple structure and easy integration etc. characteristics.^[^ ^6^, ^8^ ^]^ It is widely accepted that the change of sensor's potential signal is originated from the electrochemical reactions of analyte gas molecules and oxygen molecules at triple phase boundary (TPB: the interface of gas molecules, electrode and solid electrolyte).^[^ ^9^ ^]^ Taking acetone gas as an example, the electrochemical anode reaction and cathode reaction that occur simultaneously at TPB can be expressed as:

(1) $$O_{2}+4e^{-}\to 2O^{2-}$$

(2) $$C_{3}H_{6}O+8O^{2-}\to 3CO_{2}+3H_{2}O+16e^{-}$$

When the electrochemical reactions reach dynamic equilibrium, the mixed potential will be formed. Various sensing electrodes with different electrochemical catalytic activities lead to diverse potential signals that reflect the characteristics of analyte gas. These response signals are operable for feature engineering to achieve optimization of signal processing for discriminative gas detection.

In this work, an integrated YSZ‐based mixed potential sensor equipped with a triple‐sensing electrode array is developed for pattern recognition of VOCs gases. SEs of NiSb_2_O_6_, CuSb_2_O_6_, MgSb_2_O_6,_ and reference electrode (RE) of Pt are integrated on one YSZ substrate to form an efficient structure. Feature engineering from the perspective of data dimensionality enhancement is utilized to accentuate the distinct characteristics of every gas. Ultimately, an average classification accuracy of 98.8%, and an overall R‐squared error (R^2^) of 99.3% for concentration regression can be achieved between six VOCs gases. The integrated sensor with optimized signal processing paves the way for the replacement of bulky sensor arrays to realize discriminative detection of hazardous VOCs gases which possesses widespread applications prospect in next‐generation artificial olfaction.

## 2 Results and Discussion

### 2.1 Evaluation of Integrated YSZ‐Based Mixed Potential Sensor Equipped with Triple‐sensing Electrode Array

**Figure 1:**
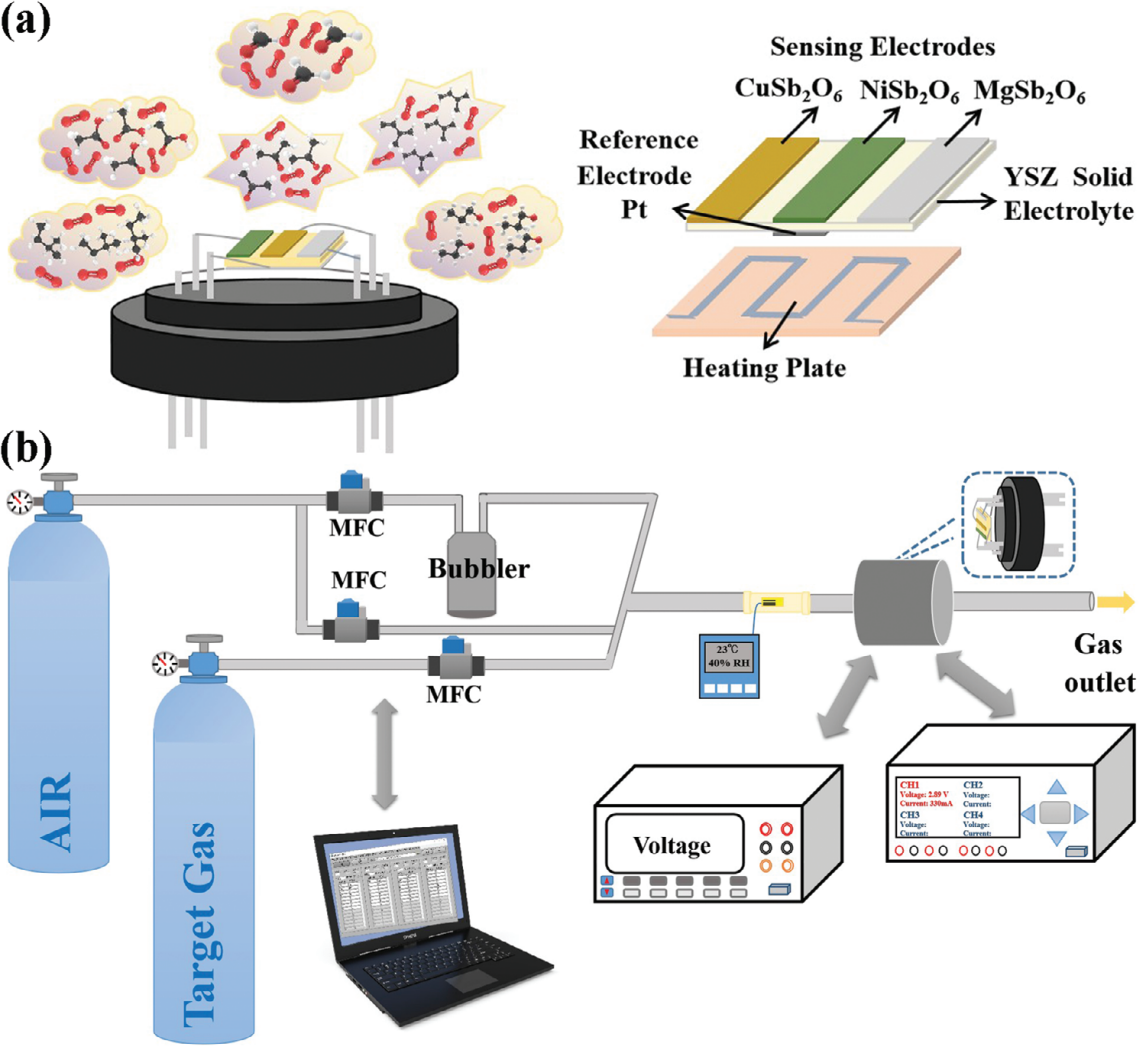
Schematic diagram of a) integrated sensor and b) dynamic measurement platform.

In this study, three sensing electrodes (NiSb_2_O_6_, CuSb_2_O_6,_ and MgSb_2_O_6_) and one shared reference electrode (Pt) are integrated on one YSZ substrate to prepare the sensor as shown in **Figure** **1**a. Its sensing performance was measured by a self‐assembled dynamic testing platform as depicted in Figure 1b. Gas sensing measurements separately to six VOCs gases of pentane, isoprene, n‐propanol, acetone, acetic acid and formaldehyde with varying concentrations were conducted. Each gas exposure experiment was repeated three times under the same condition to reduce accidental errors.

**Figure 2:**
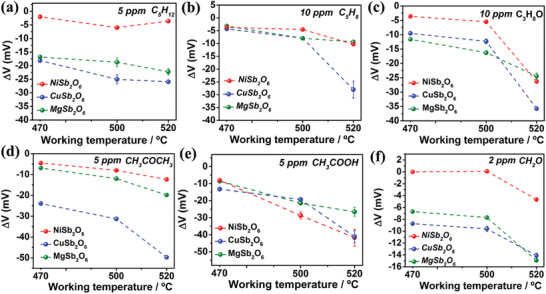
Temperature modulation response values of triple‐SE sensor.

The goal of optimizing sensor's operating temperature goes to benefit from its enhanced sensing capabilities. **Figure** **2** shows the response values of the integrated sensor with triple‐sensing electrode array to pentane, isoprene, n‐propanol, acetone, acetic acid and formaldehyde, across a range of temperatures. Notably, the vast majority of response values reach the maximum at ≈ 520 °C to six VOCs gases, except for the case of NiSb_2_O_6_‐SE to C_5_H_12_ gas. Typically, as the operating temperature increases, the greater number of activated gas molecules and increased transport rate of O^2−^ at TPB can be generated, which are propitious to elevated response values. The case of pentane may be due to the probability that further increase in temperature may result in a concomitant enhancement of gas‐phase reaction for C_5_H_12_ in NiSb_2_O_6_‐SE, thereby causing no further improvement of the response value. It is unavoidable that continual raise of operating temperature will increase the power consumption. Under comprehensive consideration, the optimal operating temperature of the device is selected to be 520 °C.

**Figure 3:**
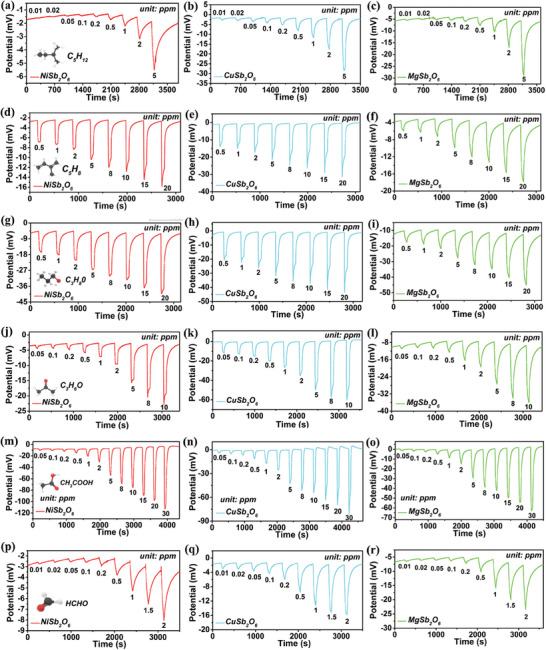
Dynamic response under different concentration range of a–c) pentane, d–f) isoprene, g–i) n‐propanol, j–l) acetone, m–o) acetic acid and p–r) formaldehyde.

**Figure 4:**
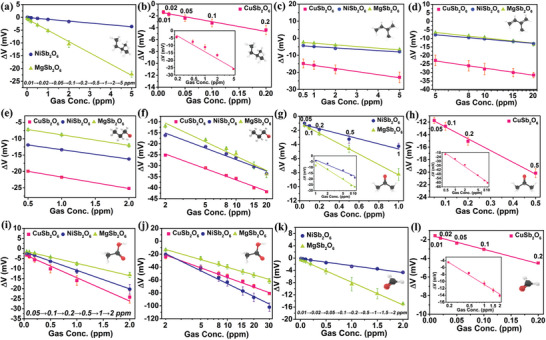
The sensitivity fitting lines to a,b) pentane, c,d) isoprene, e,f) n‐propanol, g,h) acetone, i,j) acetic acid and k,l) formaldehyde.

**Figure 5:**
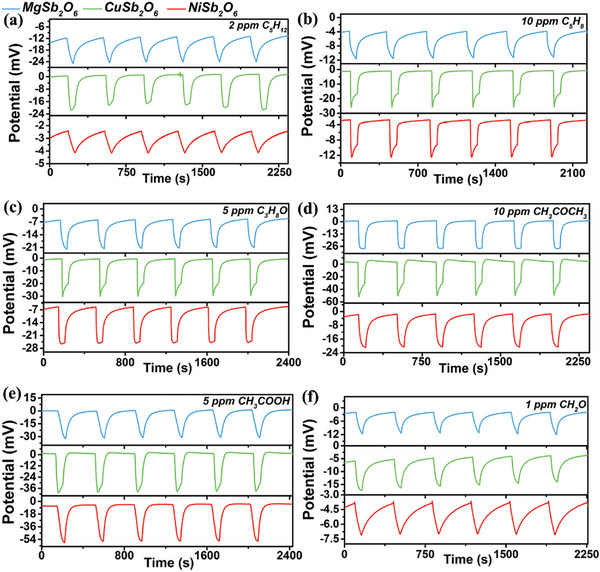
Repeatability of sensor with triple‐SE to a) pentane, b) isoprene, c) n‐propanol, d) acetone, e) acetic acid and f) formaldehyde.

In this temperature condition, the sensor can generate differentiated response patterns to various types of VOCs gases. **Figure** **3** depicts the detailed concentration gradient dynamic response of the integrated sensor to six VOCs gases. It is obvious that all the response values increase with the increase of gas concentration. Three SEs exhibit different specific response values for diverse VOCs gases. The standard deviation and the mean of corresponding response values are plotted in **Figure** **4**. A linear relationship is discernible between the response values and the gas concentrations in logarithmic coordinate or linear coordinate which is consistent with the mixed potential mechanism.^[^ ^8^, ^10^ ^]^ Repeatability of the sensor was also tested as shown in **Figure** **5**. The sensor can perform stable and repeatable potential signals and response values towards six VOCs gases. These results confirm that the sensor also possesses good repeatability.

### 2.2 Feature Engineering and Gas Classification

In order to realize qualitative gas identification and quantitative concentration prediction, machine learning is further demanded.^[^ ^2^, ^11^ ^]^ In this study, The programming language is developed by python (3.8.10) on Linux (Ubuntu 20.04 64 bit) system. Scikit‐learn is deployed to build different algorithm models. The preprocessing of data is processed by Pandas. To train different algorithm models, the dataset is validated with a training set and testing set with 70% and 30% data separately.

The response data is utilized to train algorithm model for pattern recognition. Tenfold cross‐validation is used to avoid overfitting. Machine learning algorithms such as Decision Tree (DT), Support Vector Machine with radial basis function kernel (SVM‐RBF), Support Vector Machine with linear kernel (SVM‐LIN), Logistic Regression (LR), K‐Nearest Neighbor (KNN) and Linear Discriminant Analysis (LDA) are applied for gas classification.

**Figure 6:**
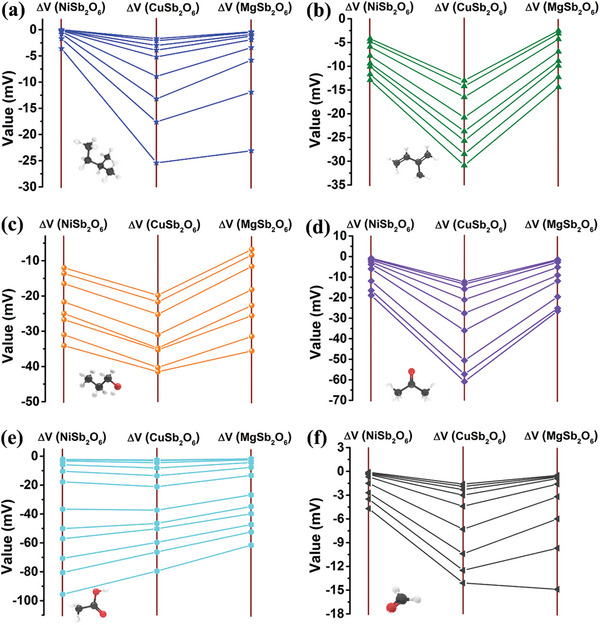
Radar plot of response values toward a) pentane, b) isoprene, c) n‐propanol, d) acetone, e) acetic acid and f) formaldehyde.

**Figure 7:**
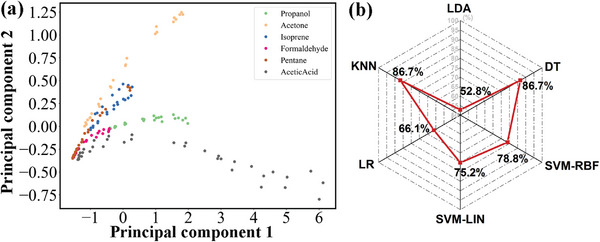
a) PCA scatter plot for six VOCs analyte gases with input vector of (S_1_, S_2_, S_3_); b) Accuracy of discrimination between six VOCs gases with different algorithms with input vector of (S_1_, S_2_, S_3_).

For the purpose of visualizing the response patterns of six VOCs gases, **Figure** **6** depicts the response values of the sensor with varying concentrations in a parallel coordinate plot. To verify the discrimination capability on the basis of response values, input vector of (S_1_, S_2_, S_3_) as described in **Table** **1** is adopted for model training. **Figure** **7**a is the scatter plot of principal component analysis (PCA) for six VOCs target gases. It can not present clear separation between the clusters representing individual gas. As shown in Figure 7b, DT, SVM‐RBF, SVM‐LIN, LR, KNN and LDA models also display poor degree of accuracy. The models merely yield the highest classification accuracy of 86.7%. The results demonstrate that it cannot distinguish well between six VOCs gases simply utilizing the response values as feature parameters. Therefore, it is crucial to further select appropriate feature parameters for more accurate classification.

**Table 1:** The features parameters definition.

Feature	Definition	Description
Group I		
S_1_	$\;\Delta V_{\mathrm{CuS}b_{2}O_{6}}=V_{\mathrm{gas}\;\mathrm{CuS}b_{2}O_{6}}-V_{\mathrm{air}\;\mathrm{CuS}b_{2}O_{6}}$	The response value with CuSb_2_O_6_‐SE
S_2_	$\;\Delta V_{\mathrm{NiS}b_{2}O_{6}}=V_{\mathrm{gas}\;\mathrm{NiS}b_{2}O_{6}}-V_{\mathrm{air}\;\mathrm{NiS}b_{2}O_{6}}$	The response value with NiSb_2_O_6_‐SE
S_3_	$\;\Delta V_{\mathrm{MgS}b_{2}O_{6}}=V_{\mathrm{gas}\;\mathrm{MgS}b_{2}O_{6}}-V_{\mathrm{air}\;\mathrm{MgS}b_{2}O_{6}}$	The response value with MgSb_2_O_6_‐SE
Group II		
$R_{1}^{1}$	$\Delta V_{\mathrm{NiS}b_{2}O_{6}}/\Delta V_{\mathrm{CuS}b_{2}O_{6}}$	First‐order response value ratio
$R_{2}^{1}$	$\Delta V_{\mathrm{MgS}b_{2}O_{6}}/\Delta V_{\mathrm{NiS}b_{2}O_{6}}$	First‐order response value ratio
$R_{3}^{1}$	$\Delta V_{\mathrm{MgS}b_{2}O_{6}}/\Delta V_{\mathrm{CuS}b_{2}O_{6}}$	First‐order response value ratio
Group III		
$R_{1}^{2}$	${\Delta V}_{\mathrm{NiS}b_{2}O_{6}}^{2}/\left(\Delta V_{\mathrm{CuS}b_{2}O_{6}}*\Delta V_{\mathrm{MgS}b_{2}O_{6}}\right)$	Second‐order response value ratio
$R_{2}^{2}$	${\Delta V}_{\mathrm{MgS}b_{2}O_{6}}^{2}/\left(\Delta V_{\mathrm{CuS}b_{2}O_{6}}*\Delta V_{\mathrm{NiS}b_{2}O_{6}}\right)$	Second‐order response value ratio
$R_{3}^{2}$	${\Delta V}_{\mathrm{CuS}b_{2}O_{6}}^{2}/\left(\Delta V_{\mathrm{NiS}b_{2}O_{6}}*\Delta V_{\mathrm{MgS}b_{2}O_{6}}\right)$	Second‐order response value ratio
Group IV		
$R_{1}^{3}$	${\Delta V}_{\mathrm{NiS}b_{2}O_{6}}^{2}/\Delta V_{\mathrm{CuS}b_{2}O_{6}}$	Third‐order response value ratio
$R_{2}^{3}$	${\Delta V}_{\mathrm{MgS}b_{2}O_{6}}^{2}/\Delta V_{\mathrm{NiS}b_{2}O_{6}}$	Third‐order response value ratio
$R_{3}^{3}$	${\Delta V}_{\mathrm{MgS}b_{2}O_{6}}^{2}/\Delta V_{\mathrm{CuS}b_{2}O_{6}}$	Third‐order response value ratio

For the sake of obtaining effective features and accentuating the distinct characteristics of every gas, feature engineering of data dimensionality enhancement is performed. Subsequently, 12 features parameters based on response values are assigned into four groups as annotated in Table 1. A preparatory parameter selection is performed to explore their importance. **Table** **2** shows the superiority of input vectors of $\left(R_{1}^{1},{\;R}_{2}^{1},{\;R}_{3}^{1}\right)$ and $\left(R_{1}^{2},{\;R}_{2}^{2},{\;R}_{3}^{2}\right)$ with the highest accuracy of 92.2% and 91.0%. However, the DT algorithm model can only provide an accuracy of 84.4% with an input vector of $\left(R_{1}^{3},{\;R}_{2}^{3},{\;R}_{3}^{3}\right)$. The improvement of model accuracy indicates that the expanded feature parameters play a critical role in gas pattern recognition.

**Table 2:** Average accuracy of tenfold cross‐validation of discrimination between six VOCs analyte gases under diverse algorithms with different input vectors.

Input vector	Accuracy					
	DT	SVM‐RBF	SVM‐LIN	LR	KNN	LDA
(S_1_, S_2_, S_3_)	86.7%	78.8%	75.2%	66.1%	86.7%	52.8%
$\left(R_{1}^{1},{\;R}_{2}^{1},{\;R}_{3}^{1}\right)$	89.2%	92.2%	89.7%	84.9%	89.8%	77.1%
$\left(R_{1}^{2},{\;R}_{2}^{2},{\;R}_{3}^{2}\right)$	89.1%	90.3%	91.0%	85.0%	84.3%	75.3%
($R_{1}^{3},{\;R}_{2}^{3},{\;R}_{3}^{3})$	84.4%	74.0%	60.0%	61.3%	79.5%	37.7%
$\left(S_{1},\;S_{2},\;S_{3},{\;R}_{1}^{1},{\;R}_{2}^{1},{\;R}_{3}^{1}\right)$	93.3%	98.2%	98.8%	97.6%	98.2%	86.7%
$(S_{1},\;S_{2},\;S_{3},R_{1}^{1},{\;R}_{2}^{1},{\;R}_{3}^{1},$ $R_{1}^{2},{\;R}_{2}^{2},{\;R}_{3}^{2})$	95.8%	97.6%	98.2%	98.2%	97.6%	90.4%
$(S_{1},\;S_{2},\;S_{3},{\;R}_{1}^{1},{\;R}_{2}^{1},{\;R}_{3}^{1},$ $R_{1}^{2},{\;R}_{2}^{2},{\;R}_{3}^{2},{\;R}_{1}^{3},{\;R}_{2}^{3},{\;R}_{3}^{3})$	95.8%	97.6%	98.2%	97.6%	96.9%	97.0%
$\left(R_{1}^{1},{\;R}_{2}^{1},{\;R}_{3}^{1},{\;R}_{1}^{2},{\;R}_{2}^{2},{\;R}_{3}^{2}\right)$	92.8%	90.4%	90.4%	88.6%	90.4%	77.2%
$(R_{1}^{1},{\;R}_{2}^{1},{\;R}_{3}^{1},{\;R}_{1}^{2},{\;R}_{2}^{2},{\;R}_{3}^{2},$ $R_{1}^{3},{\;R}_{2}^{3},{\;R}_{3}^{3})$	95.1%	96.4%	94.6%	92.8%	95.8%	88.6%
$\left(R_{1}^{2},{\;R}_{2}^{2},{\;R}_{3}^{2},{\;R}_{1}^{3},{\;R}_{2}^{3},{\;R}_{3}^{3}\right)$	93.4%	94.6%	91.0%	89.2%	94.6%	79.9%

Additionally, an analysis about further enhancing the classification accuracy also needs to be discussed. Owing to the elevated accuracy based on elementary feature selection, analysis based on the more combinations of feature parameters is carried out. From the comparison in Table 2, it shows that the accuracy based on different combinations between extended feature parameters is generally improved. By comparison, the feature parameters of Group I and Group II contribute to higher classification accuracy. Considering the influence of features of Group I and Group II, the input vectors of $\left(S_{1},\;S_{2},\;S_{3},{\;R}_{1}^{1},{\;R}_{2}^{1},{\;R}_{3}^{1}\right)$, $\left(S_{1},\;S_{2},\;S_{3},{\;R}_{1}^{1},{\;R}_{2}^{1},{\;R}_{3}^{1},{\;R}_{1}^{2},{\;R}_{2}^{2},{\;R}_{3}^{2}\right)$ and ($S_{1},\;S_{2},\;S_{3},{\;R}_{1}^{1},{\;R}_{2}^{1},{\;R}_{3}^{1},{\;R}_{1}^{2},{\;R}_{2}^{2},{\;R}_{3}^{2},{\;R}_{1}^{3},{\;R}_{2}^{3},{\;R}_{3}^{3}$) can display better accuracy of 98.8%, 98.2%, and 98.2%. Table 2 exhibits the optimal accuracy of 98.8% which demonstrates that data dimensionality enhancement improves the accuracy of pattern recognition. The metrics in **Table** **3** also prove the high success rate of classification by the SVM‐LIN classifier toward six VOCs gases. All data is based on the spike signals within 60 s that avoids waiting for the signal to stabilize or recover for a complete response and recovery process. It could improve detection efficiency with the spike signal of high amplitude which facilitates rapid discriminative detection of hazardous VOCs gases.

**Table 3:** The performance metrics of classification toward six VOCs gases based on SVM‐LIN classifier.

Gas type	Precision (%)	Recall (%)	F1 score (%)
N‐propanol	100	100	100
Acetone	100	100	100
Isoprene	100	100	100
Formaldehyde	100	96.3	98.1
Pentane	96.4	100	98.2
Acetic acid	100	100	100

### 2.3 Concentration Prediction

**Figure 8:**
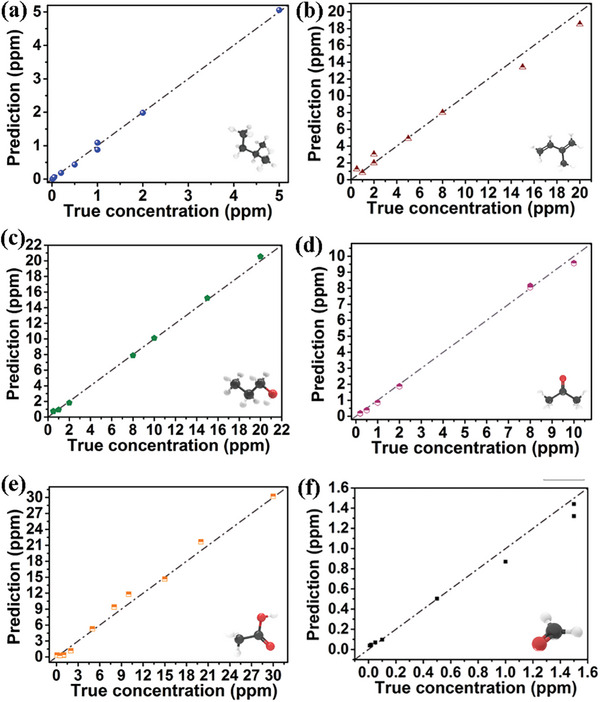
Relationships of predicted gas concentrations versus true concentrations for six VOCs gases through polynomial regression model.

Further quantitative prediction of gas concentrations is studied with SVM‐LIN, SVM‐RBF and polynomial regression models. As compared in **Table** **4**, polynomial regression model exhibits the best concentration regression results among the three algorithms. In detailed prediction performance from validation data for six VOCs gases of **Figure** **8**, the closer the point is to the dotted line, the more accurate quantitative prediction can be proved. It can be seen that most of prediction results from polynomial regression model are close to the real concentrations. The performance of the model is evaluated using mean absolute error (MAE), mean squared error (MSE), and R^2^ as summarized in **Table** **5**. The R^2^ scores are as high as 99.9%, 97.8%, 99.9%, 99.8%, 99.0% and 97.9% for C_5_H_12_, C_5_H_8_, C_3_H_8_O, C_3_H_6_O, C_2_H_4_O_2_ and CH_2_O. Furthermore, the overall regression results display MAE, MSE and R^2^ scores of 0.316, 0.314, and 99.3%. **Table** **6** displays the accuracy comparison of gas identification in previous works and this study. It can demonstrate the effectiveness of the integrated YSZ‐based mixed potential sensor equipped with a triple‐sensing electrode array for quantitative discriminative detection toward six VOCs gases.

**Table 4:** The prediction performance under corresponding algorithm models.

Metrics	SVM‐LIN	SVM‐RBF	Polynomial Regression
MAE	0.775	0.490	0.316
MSE	1.489	0.890	0.314
R^2^	95.5%	97.7%	99.3%

**Table 5:** The detailed prediction performances under polynomial regression model.

Metrics	Pentane	Isoprene	N‐propanol	Acetone	Acetic acid	Formaldehyde	Six VOCs gases
MAE	0.043	0.643	0.218	0.121	0.749	0.053	0.316
MSE	0.003	0.794	0.067	0.029	0.885	0.006	0.314
R^2^	99.9%	97.8%	99.9%	99.8%	99.0%	97.9%	99.3%

**Table 6:** The performance comparison between previously reported literature and this work.

Type of sensor/sensor array	Sensing Material	Element number	Number of classified gases	Classification accuracy (%)	Quantification	Reference
YSZ‐based integrated sensor array	NiSb_2_O_6_; CuSb_2_O_6_; MgSb_2_O_6_	3	6	98.8%	R^2^: 99.3%	This work
Semiconductor‐based sensor array	SnO_2_; In_2_O_3_; WO_3_ and CuO with and without Au nanoparticle decoration	8	6	98.1%	average error: 10.15%.	[3a]
	In_2_O_3_ with different Ga doping/alloying levels	4	6	92.85%	Accuracy: 99.14%	[12]
	(Commercial sensors)	10	6	94.4%	**/**	[4e]
	N‐doped graphene quantum dots; MoS_2_; Au; Pd	4	5	97%	**/**	[4a]
ZnO‐based MEMS sensor array	ZnO and ZnO with additives	6	4	97.9%	R^2^: 0.975	[13]
Graphene‐based sensor array	Functionalized Graphene	108	5	98%	**/**	[3b]
Polymer‐based sensor array	paper‐based; poly(2‐acrylamido‐2‐methyl‐1‐propanesulfonic acid); N‐3‐ (dimethylamino)propyl methacrylamide (DMAPMAm) and methoxyethyl methacrylate (MEMA) (PD‐co‐M), and diethylamine (DEA)	3	3	**/**	**/**	[14]
Polymer‐based sensor array	Graphene and polyaniline composite	4	2	100%	R^2^: over 99%	[15]
Polymer‐based sensor array	polymer‐graphene nanoplatelet composite	12	5	99%	**/**	[16]
Polymer‐based sensor array	Poly(styrene); Poly(caprolactone); Poly(ethylene‐co‐vinyl acetate); Poly(vinyl chloride); Nafion	7	5	**/**	**/**	[17]
Polymer‐based sensor	Pt	1	2	**/**	**/**	[18]
Polymer‐based sensor	Polypyrrole	1	3	**/**	**/**	[19]
Polymer‐based sensor	3D sulfonated RGO hydrogel	1	4	**/**	**/**	[20]

## 3 Conclusion

In this paper, we propose an integrated YSZ‐based mixed potential sensor equipped with a triple‐sensing electrode array combining with feature engineering for discriminative detection of six VOCs gases. Relatively low concentration ranges of pentane, isoprene, n‐propanol, acetone, acetic acid, and formaldehyde can be detected with distinctive response values. On the basis of feature engineering from spike response signal, data dimensionality enhancement and optimized selection of features are further adopted. Eventually, elevated classification accuracy of 98.8% between six VOCs gases is achieved, resulting from that feature engineering allows for amplification of the distinct characteristics of every gas. In addition, the sensor is capable of predicting the untrained dataset to verify the ability of quantitative prediction. The overall R^2^ error for concentration regression can achieve 99.3% toward six VOCs gases. The purpose of replacing large‐size sensor array with efficient integrated sensor can also be realized, which will have important practical significance and broad application prospects in next‐generation electronic nose for hazardous gases.

## 4 Experimental Section

### Design of Integrated Sensor with Triple‐Sensing Electrode Array

Three sensing electrodes (NiSb_2_O_6_, CuSb_2_O_6,_ and MgSb_2_O_6_) and one shared reference electrode (Pt) were integrated on one YSZ substrate. The synthetic methods of sensing materials can be obtained from the previous works where their characterization results have been detailed.^[^ ^8^ ^]^ The heating plate was bonded with the RE side of the YSZ substrate by an inorganic adhesive. Eventually, the sensor was connected to a hexagonal tube socket for testing. Through applying current to the heating plate, the desired working temperature of the sensor could be provided. The exact temperature value was standardized by the FLIR T250 thermal infrared imager.

### Measurement Platform and Testing Process

The gas flow rate through the chamber was set by digital mass flow controllers (MFCs). Six analyte gases with varying concentrations were modulated by controlling the flow ratio of dry synthetic air, humid synthetic air, and standard gas. In the gas circuit, the humidity was set at ≈40% RH, and the temperature is retained within the range of 23 ± 1 °C.

During the testing procedure, the potential differences between the three SEs and RE were measured as voltage signals every second. The maximal difference of voltage signal between analyte gas and air is defined as response value (ΔV  = V_gas_  − V_air_).

## Conflict of Interest

The authors declare no conflict of interest.

## Data Availability

The data that support the findings of this study are available from the corresponding author upon reasonable request.
